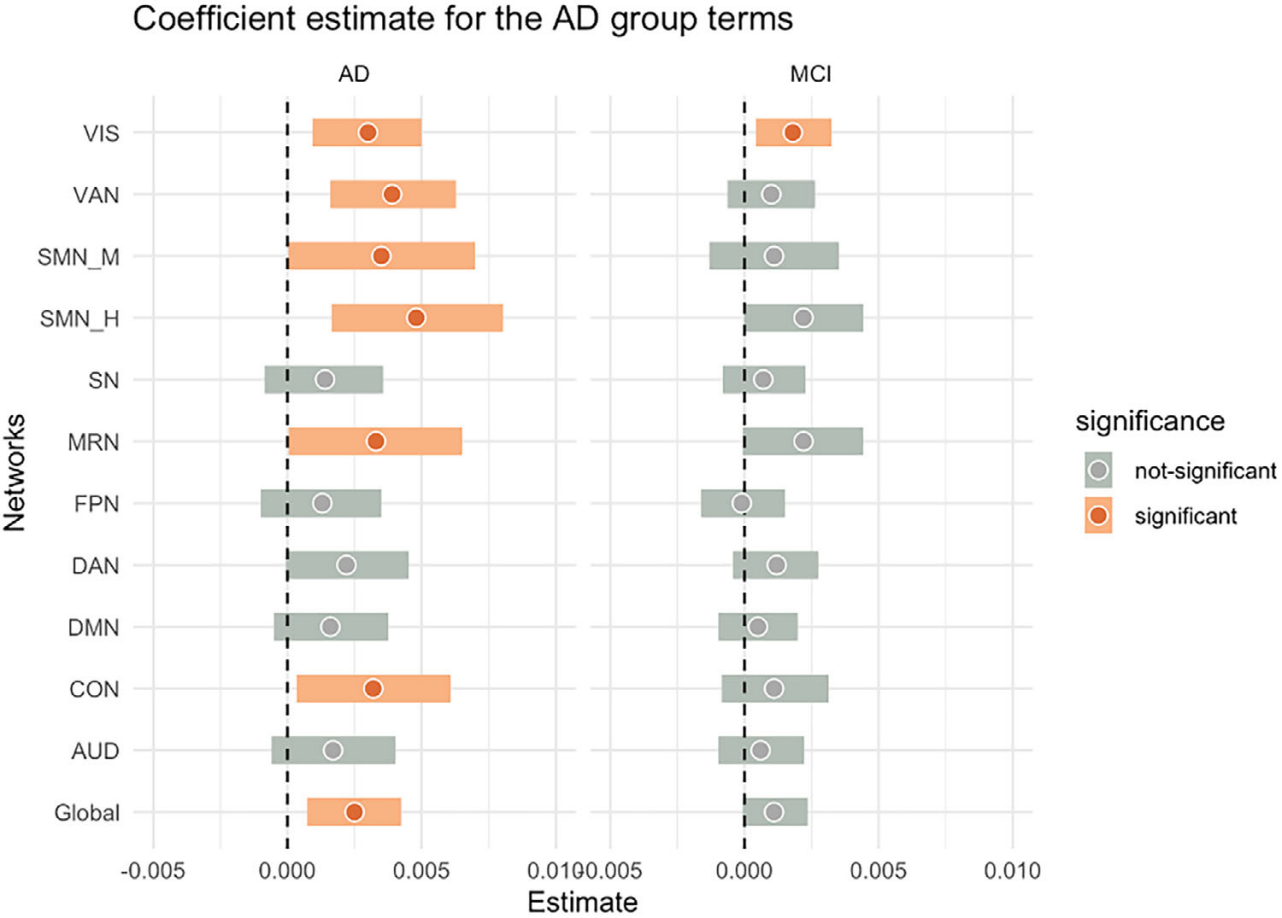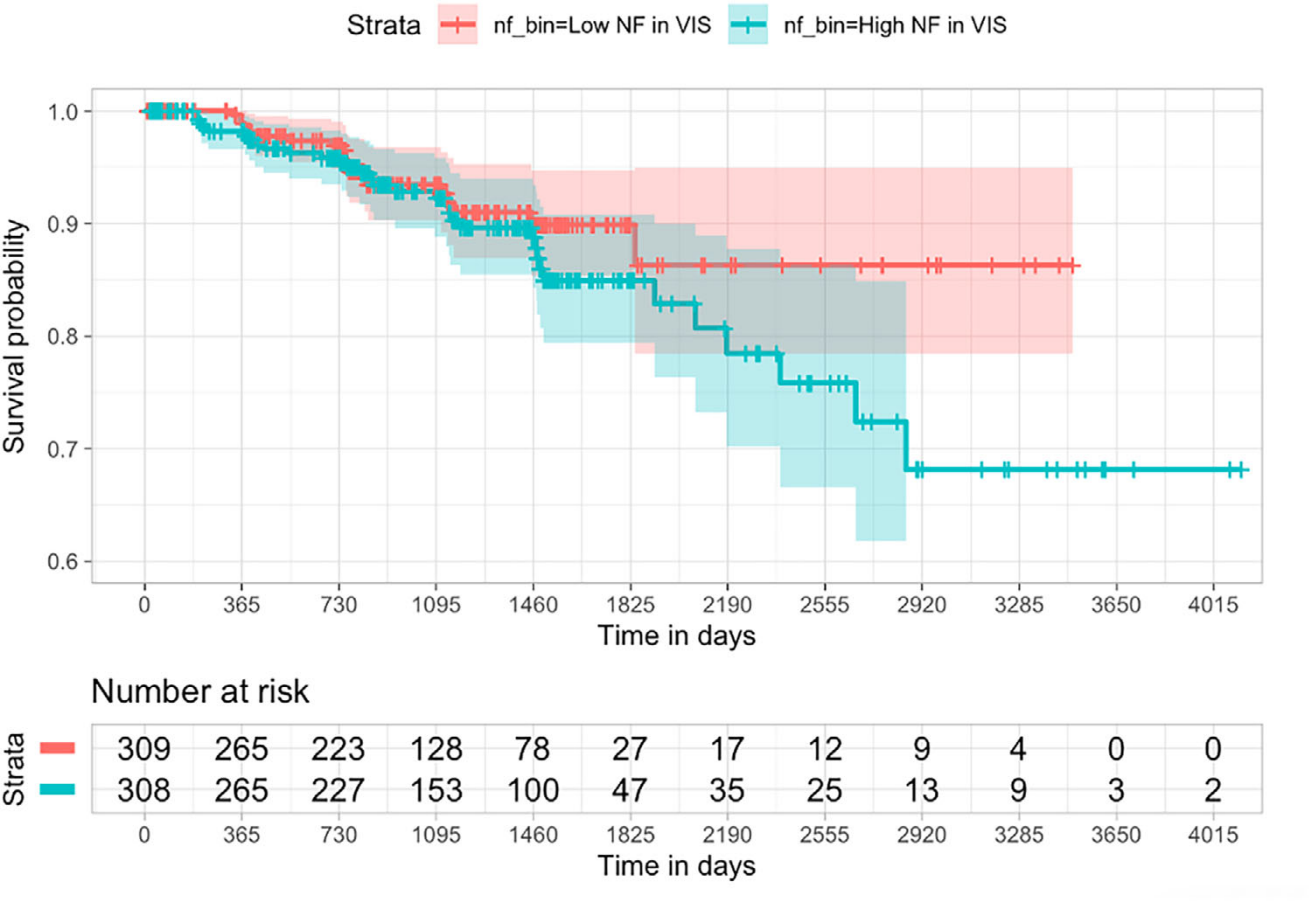# Neural flexibility as a promising biomarker for Alzheimer’s Disease

**DOI:** 10.1002/alz.095766

**Published:** 2025-01-09

**Authors:** Eleanna Varangis, Jun Liu, Yiqi Miao, Xi Zhu, Yaakov Stern, Seonjoo Lee

**Affiliations:** ^1^ School of Kinesiology, Michigan University, Ann Arbor, MI USA; ^2^ New York State Psychiatric Intstitute, New York, NY USA; ^3^ Columbia University, New York, NY USA; ^4^ New York State Psychiatric Institute, New York, NY USA; ^5^ Columbia University Irving Medical Center, New York, NY USA; ^6^ Cognitive Neuroscience Division, Columbia University, New York, NY USA; ^7^ Mailman School of Public Health, Columbia University, New York, NY USA

## Abstract

**Background:**

Neural flexibility (NF) during tasks was associated with cognitive aging, while that during rest was not associated with aging and cognition in a healthy aging population. However, NF has not been studied in AD. We aim to evaluate whether AD is associated with alterations in NF and probe its predictive utility for AD conversion.

**Method:**

The study included 862 older adults with valid resting‐state fMRI data: 461 who are cognitively normal (CN, 53.5%), 294 with Mild cognitive impairment (MCI, 34.1%), and 107 with Alzheimer’s Disease (AD, 12.4%) from the Alzheimer’s Disease Neuroimaging Initiative (ADNI). The MRI images were processed using fMRIPrep, and the mean time series of each node was extracted according to the Power Atlas parcellation scheme. We performed dynamic community detection based on generalized Louvain methods. We defined the NF of a node as the number of times that a node changed its community assignment across the sliding windows, normalized by the total number of possible changes and computed global NF (GNF) and network‐level NF. We performed linear mixed models on NF to explore the effect of AD group indicators on NF controlling for the age at scan, gender, education level, and the random intercept of site. We then evaluated the dementia transition of neural flexibility using survival analysis. In the non‐demented participants at baseline, we performed Cox‐proportional hazard regression analysis with each NF.

**Results:**

NF is significantly higher in AD group than the CN group on global, CON, MRN, SMN‐H, SMN‐M, VAN, and VIS networks (Figure 1); neural flexibility is significantly higher in the MCI group than the CN group in the VIS network (Figure 1). Among n = 670 non‐demented participants at baseline, n = 53 (8.6%) participants converted to dementia during the follow‐ups. Higher neural flexibility in VIS was positively associated with AD transition (HR = 1.323, 95%CI 1.002 to 1.747, p = 0.049, per 1 standard deviation in NF, Figure 2), controlling for age, gender, and education.

**Conclusion:**

We found that NF during resting was higher in AD patients and predicted dementia transition. Thus, NF can be a valuable biomarker of AD, and more validation and mechanistic studies need to be performed.